# Significance of Antisolvents on Solvation Structures Enhancing Interfacial Chemistry in Localized High-Concentration Electrolytes

**DOI:** 10.1021/acscentsci.2c00791

**Published:** 2022-08-31

**Authors:** Yanzhou Wu, Aiping Wang, Qiao Hu, Hongmei Liang, Hong Xu, Li Wang, Xiangming He

**Affiliations:** † Institute of Nuclear and New Energy Technology, Tsinghua University, State Key Laboratory of Automotive Safety and Energy, Beijing 100084, China

## Abstract

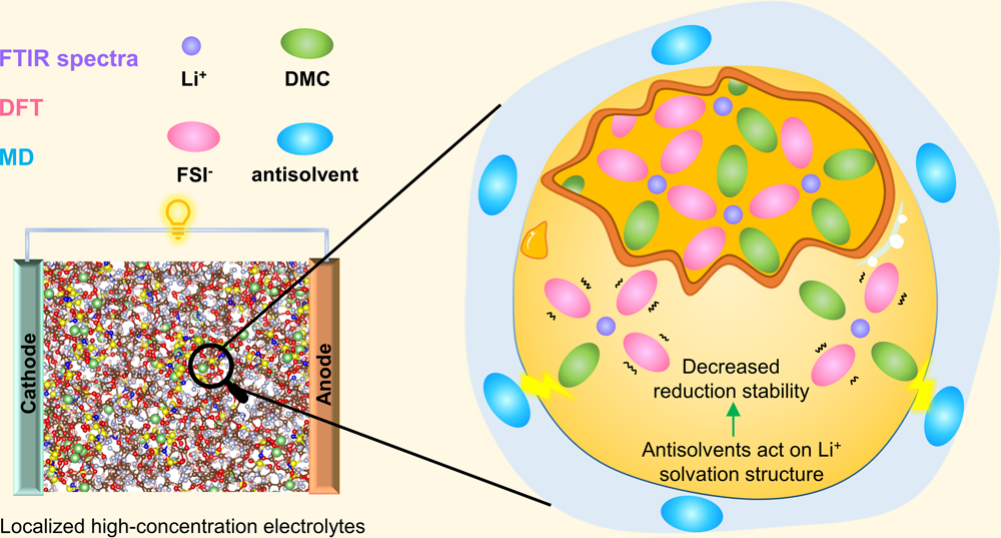

Localized high-concentration electrolytes (LHCEs) provide a new way to expand multifunctional electrolytes because of their unique physicochemical properties. LHCEs are generated when high-concentration electrolytes (HCEs) are diluted by antisolvents, while the effect of antisolvents on the lithium-ion solvation structure is negligible. Herein, using one-dimensional infrared spectroscopy and theoretical calculations, we explore the significance of antisolvents in the model electrolyte lithium bis(fluorosulfonyl)imide/dimethyl carbonate (LiFSI/DMC) with hydrofluoroether. We clarify that the role of antisolvent is more than dilution; it is also the formation of a low-dielectric environment and intensification of the inductive effect on the C=O moiety of DMC caused by the antisolvent, which decrease the binding energy of the Li^+^···solvent and Li^+^···anion interactions. It also has beneficial effects on interfacial ion desolvation and Li^+^ transport. Furthermore, antisolvents also favor reducing the lowest unoccupied molecular orbital (LUMO) energy level of the solvated clusters, and FSI^–^ anions show a decreased reduction stability. Consequently, the influence of antisolvents on the interfacial chemical and electrochemical activities of solvation structures cannot be ignored. This finding introduces a new way to improve battery performance.

## Abstract 2

The antisolvent in localized high-concentration electrolytes plays a role in more than dilution. Antisolvents change the Li^+^ solvation structure through intermolecular interactions.

## 1 Introduction

Lithium-ion batteries (LIBs) are ideal for the future development of energy storage devices due to their high energy densities.^1,2^ To constantly improve the performance of LIBs, such as by achieving a higher specific energy, wider temperature range, higher rate capability, and longer cycle life, efforts have been made to explore new electrode materials or battery designs. The electrode materials determine the theoretical performance of the batteries, while the electrolyte has a strong influence on the deliverable performance of the electrode materials. However, most new battery chemistries are beyond the applicability of commercial electrolytes (typically lithium hexafluorophosphate (LiPF_6_) carbonate-based).^3^ Therefore, advanced electrolytes, both for new battery chemistries and for improving the performance of existing batteries, are of considerable interest.

The impact of electrolytes on battery performance is mainly manifested in the following ways. First, the interfacial compatibility with the electrode material, including wettability and chemical/electrochemical stability, affects the specific energy and rate capacity of the battery. Second, the ion transfer capacity affects the power performance of the battery. Third, the physical properties of the fluid, including viscosity, temperature adaptability, flammability, and so on, play a role. Generally, free solvent molecules affect the physical properties of the electrolytes, while solvated solvent molecules play an important role in the interfacial reactions with electrode materials, either chemically or electrochemically. However, in conventional homogeneous carbonate-based electrolytes with a concentration of approximately 1 M, there is an exchange between the solvated solvent and free solvent, resulting in the coupling of bulk and interfacial properties.

Commonly, the regulation of electrolyte properties includes the introduction of an additive, which modifies either the interface reaction or the bulk properties,^4−6^ or concentration optimization.^7,8^ In recent years, the proposed localized high-concentration electrolytes (LHCEs) have decoupled the free solvent from the solvated solvent in terms of species and properties, providing insight into next-generation batteries with high energy density and excellent cyclic stability.^9,10^ LHCEs are multiphase microstructures that have been investigated by molecular dynamics (MD) simulations.^11,12^ In detail, the salt:solvent ratio in LHCEs is as high as that in superconcentrated electrolytes, which are characterized by the high participation of anions in the solvated sheath. The solvents as the free phase are antisolvents with low viscosity, low or nonflammability, and high electrochemical stability; for example, hydrofluoroethers (HFEs) are the most commonly used antisolvents. LHCEs have been proven by many studies to favor the formation of an inorganic-rich solid-electrolyte interphase (SEI)^13,14^ and cathode electrolyte interphase (CEI).^15−17^ This is why LHCEs are the most promising candidates for stabilizing high-voltage cathodes,^18−20^ enabling highly reversible Li metal anodes^21,22^ and stabilizing lithium polysulfides to achieve the high capacity and cycling ability of Li–S batteries.^23,24^ Moreover, the introduction of an antisolvent is an important strategy to reduce viscosity, improve low temperature,^25,26^ and promote nonflammable^14,27,28^ properties of electrolytes. Therefore, the selection of appropriate antisolvents can offer new possibilities for the development of high-performance LMBs.

The intermolecular interaction between solvent and antisolvent molecules is expected to be strong according to their mutual solubility. It can be understood that solvent molecules in the solvation sheath will be affected by the antisolvent due to their strong interaction. This is why LCHEs with different physical and chemical properties can be prepared by only changing the molecular structure of the antisolvents.^29,30^ Recently, many works have demonstrated that antisolvents do not participate in the solvation sheath within Li^+^ by MD simulations and spectroscopy. Nevertheless, the influence of antisolvent on the solvation structures has been observed.^30−33^ For instance, Li et al. have utilized the Raman spectra, nuclear magnetic resonance (NMR) spectra, and MD simulations to study the solvation structures of Na^+^.^32^ They find that the higher 1*H*,1*H*,5*H*-octafluoropentyl-1,1,2,2-tetrafluoroethyl ether (OTE) additions can enhance the coordination of FSI^–^ anions with Na^+^, due to the dissolution of 1,2-dimethoxyethane (DME) in OTE. Huang et al. report that the low dielectric environment afforded by antisolvent can enhance the interaction between anion and Li^+^ in DME-based LHCEs.^30^ However, the mechanism of how an antisolvent acts on the solvation structure and changes the electrochemical reactivity of the solvation structure remains unclear and inadequate. To obtain theoretical guidance and further the development of high-performance LHCEs, here, we investigate the solvation behavior of various LHCEs using vibrational spectroscopy, density functional theory (DFT), and molecular dynamics (MD) simulations. Specifically, antisolvents affect the solvation structure through intermolecular interactions with the solvent and consequently optimize the stability of electrolyte components. The degree of influence depends on the type of antisolvent. In addition, antisolvents enable the Li^+^ desolvation process and promote the Li^+^ transport in LHCEs.

## 2 Experimental Section

### 2.1 Electrolyte Preparation

Lithium bis(fluorosulfonyl)imide (LiFSI) and dimethyl carbonate (DMC) were purchased from DoDoChem with 98% purity. 1,1,2,2-Tetrafluoroethyl-2,2,2-trifluoroethyl ether (TFETFE) (99.8%) and 1,1,2,2-tetrafluoroethyl-2,2,3,3-tetrafluoropropyl ether (TTE) (99.9%) were purchased from Sinochem Lantian Co., Ltd. They were dehydrated with 4 Å molecular sieves before they were used to dilute the electrolyte. LiFSI was dissolved in DMC to form a high-concentration (4.5 M) electrolyte (HCE), with a DMC/LiFSI molar ratio of 1.5:1. A localized high-concentration electrolyte (LHCE) was prepared by adding TFETFE or TTE into the HCE (4.5 M LiFSI/DMC solution) with a molar ratio of 1:1.5:1.5. All electrolytes were prepared in a glovebox filled with argon (<1 ppm of H_2_O).

### 2.2 FTIR Experiment

Fourier transform infrared (FTIR) spectral data of the electrolyte samples were obtained by using a Nicolet 6700 FTIR spectrometer (Thermo Electron) at room temperature. To remove the interference of H_2_O and CO_2_, nitrogen was blown through the FTIR spectrometer and sample chamber. All FTIR spectra were fitted by the Voigt function consisting of Gaussian and Lorentzian functions in Origin95 software.

### 2.3 Computational Details

DFT calculations were performed by using Gaussian 16 software, at the B3LYP level of theory using the 6-311++G (d, p) basis set.^34,35^ Simultaneously, geometry optimization and frequency calculations were performed with the universal solvation model of SMD under the solvation effect with an appropriate dielectric constant.^36^ The binding energy (*E*_*b*_) between solvent/anion and Li^+^ or between solvent was calculated based on the following equation
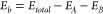
1where *E*_*total*_ is the total energy of complexes A−B, and *E*_*A*_ and *E*_*B*_ denote the energy of components A and B, respectively. The analysis of electrostatic potential was realized via Gaussian16^37^ and Multiwfn.^38^

The solvation structures of electrolytes were simulated by MD simulations, which were performed using the Large Scale Atomic/Molecular Massively Parallel Simulator (LAMMPS) code.^39^ This program has been used successfully in the field of lithium-ion batteries.^11,40,41^ General Amber force fields parameters of the solvent molecules^42,43^ were generated by the ANTECHAMBER program in AmberTools. The force field parameters of Li^+^ and FSI^–^ referred to previous publications.^44,45^ The initial atomic coordinate files were created utilizing Packing Optimization for MD Simulations (PACKMOL)^46^ and were further utilized to generate the topologies of the electrolyte systems using Moltemplate.^47^ Long-range Coulombic interactions were handled by the particle-particle particle-mesh (PPPM) method. van der Waals (vdW) interactions were described under a 12–6 Lennard–Jones interaction model, and the cutoff is set to 10 Å. The number ratio of LiFSI to DMC was 200:300 in all simulation boxes, and the number of antisolvent was 300. To eliminate the unreasonable configuration in the initial structure as much as possible, a conjugated-gradient energy minimization scheme with a convergence criterion of 1.0 × 10^–8^ was employed for the initial configuration. First, the simulation box was equilibrated for 1 ns under NPT conditions at 330 K with the aim of further relaxing the initial configuration of the system. Then, the system was rapidly cooled to 298 K and relaxed for another 5 ns. Finally, 10 ns long NVT runs were conducted under Nose-Hoover thermostats. The electrolyte structure was visualized made by using VMD^48^ and VESTA.^49^

## 3 Results and Discussion

### 3.1 Solvation Structure of Different LHCEs Characterized with FTIR Spectroscopy

**Figure 1:**
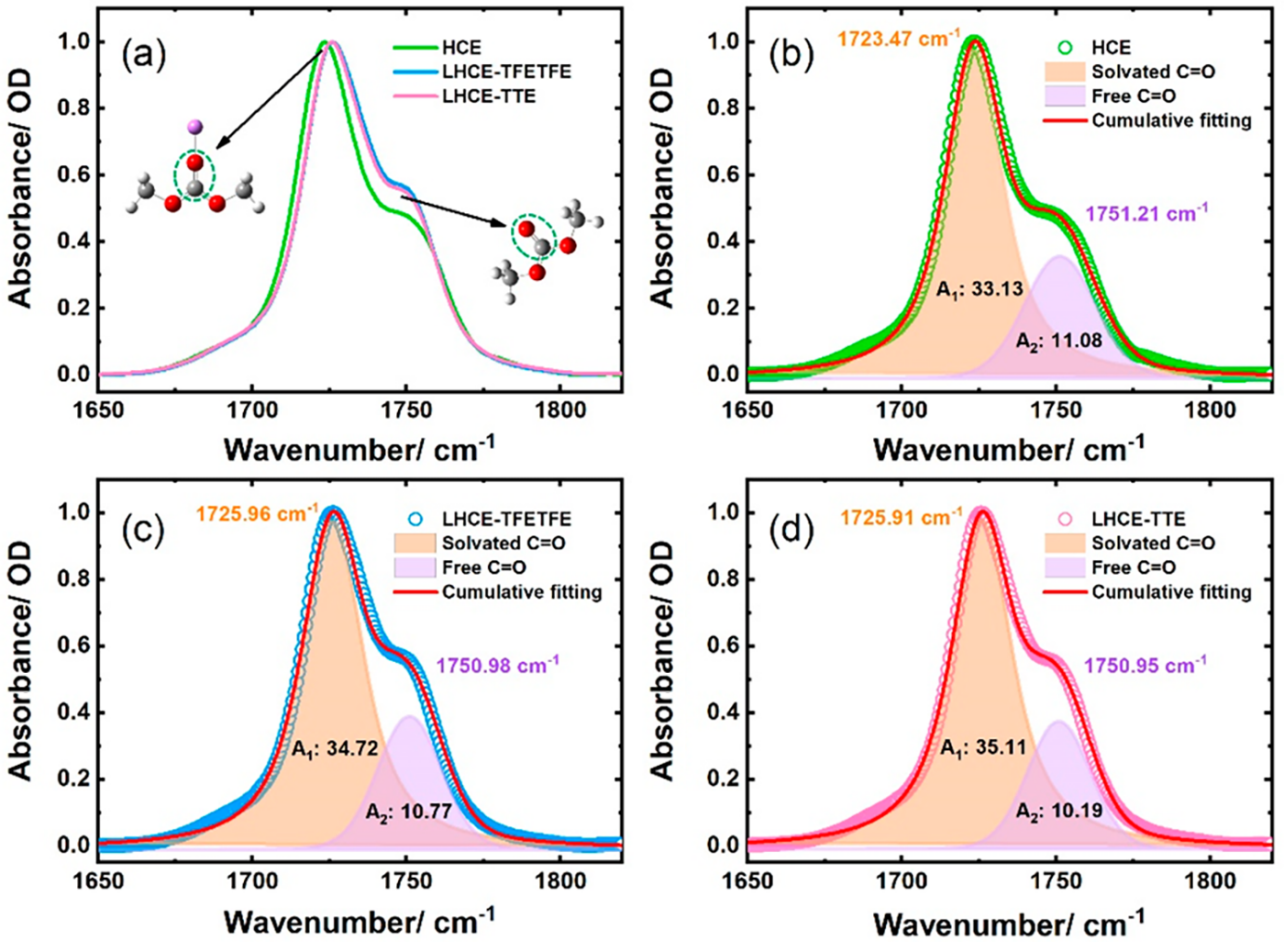
FTIR spectra of DMC in electrolytes. (a) Comparison of the C=O vibration modes in different electrolytes; (b–d) fitting spectra with the Voigt function in HCE, LHCE–TFETFE, and LHCE–TTE, respectively.

In LHCEs, the antisolvent does not coordinate with Li^+^ in the first solvated sheath and separates the characteristic 3D solvation structures of the high-concentration electrolyte into local clusters.^26^ To further reveal the effects of antisolvents on the Li^+^ solvation structures of LHCEs, three model electrolytes are investigated in this study: LiFSI/DMC (HCE, 4.5 M), LiFSI/DMC/TFETFE (LHCE–TFETFE, 1:1.5:1.5, *n*:*n*:*n*) and LiFSI/DMC/TTE (LHCE–TTE, 1:1.5:1.5, *n*:*n*:*n*). First, FTIR spectroscopy is used to gain insight into the solution structures of the electrolytes. The IR-active C=O group is sensitive to structural changes as observed in the region of 1650–1850 cm^–1^. Figure 1a shows the normalized FTIR spectra of the C=O stretching vibration of DMC solvent. For LiFSI/DMC (4.5 M) electrolyte, the FTIR spectrum shows two main peaks centered at approximately 1723 and 1751 cm^–1^. The high-frequency peak corresponds to the C=O stretching mode of free DMC, while the low-frequency band at 1723.5 cm^–1^ results from the coordinated DMC and is denoted as solvated C=O. With the addition of antisolvents, the solvated C=O band is blueshifted, and the intensity of free C=O is seemingly increased. This could be attributed to the intermolecular interactions between solvated DMC and antisolvents leading to changes in solvation structures, as revealed in Figure 1(b–d).

To evaluate the effect of antisolvent on solvation structures, the peaks of free and solvated C=O are fitted by the Voigt function. The vibrational parameters are also listed in Table 1. Here, we assume that the IR sensitivity for each band is equivalent. As previously reported, the number of coordinated and uncoordinated solvents is proportional to the integral area of the FTIR spectrum.^50^ The relative areas (*R*) are described in eq 2, which is used to estimate the degree of DMC participating in the Li^+^ solvation structure^51^
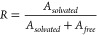
2where *A*_*solvated*_ and *A*_*free*_ are the integrated area intensities of the vibrational bands for solvated C=O and free C=O bands, respectively. The peak area of coordinated solvent is 74.9% for LiFSI/DMC (4.5 M) electrolyte. For the LHCEs, the absorption peak area of solvated C=O increases slightly, while the peak for free C=O shows an opposite trend. The peak areas of coordinated DMC for LHCE–TFETFE and LHCE–TTE are 76.3 and 77.5%, respectively. Based on the FTIR results, it is plausible that more DMC coordinates with Li^+^ in LHCEs than in HCE. Therefore, the interaction between DMC and antisolvent should be considered as much as possible in LHCEs.

**Table 1:** Fitting Parameters of the C=O Stretching Modes in the Experimental FTIR Spectra^a^

Sample	ω_*solvated*_	*A*_*solvated*_	ω_*free*_	*A*_*free*_	*R*
4.5M	1723.47	33.13	1751.29	11.08	74.9%
LHCE–TFETFE	1725.96	34.72	1750.98	10.77	76.3%
LHCE–TTE	1725.91	35.11	1750.95	10.19	77.5%

a The vibrational frequency (ω, in cm^–1^), integral area (*A*), and relative area of coordinated solvent (*R*) are given.

**Figure 2:**
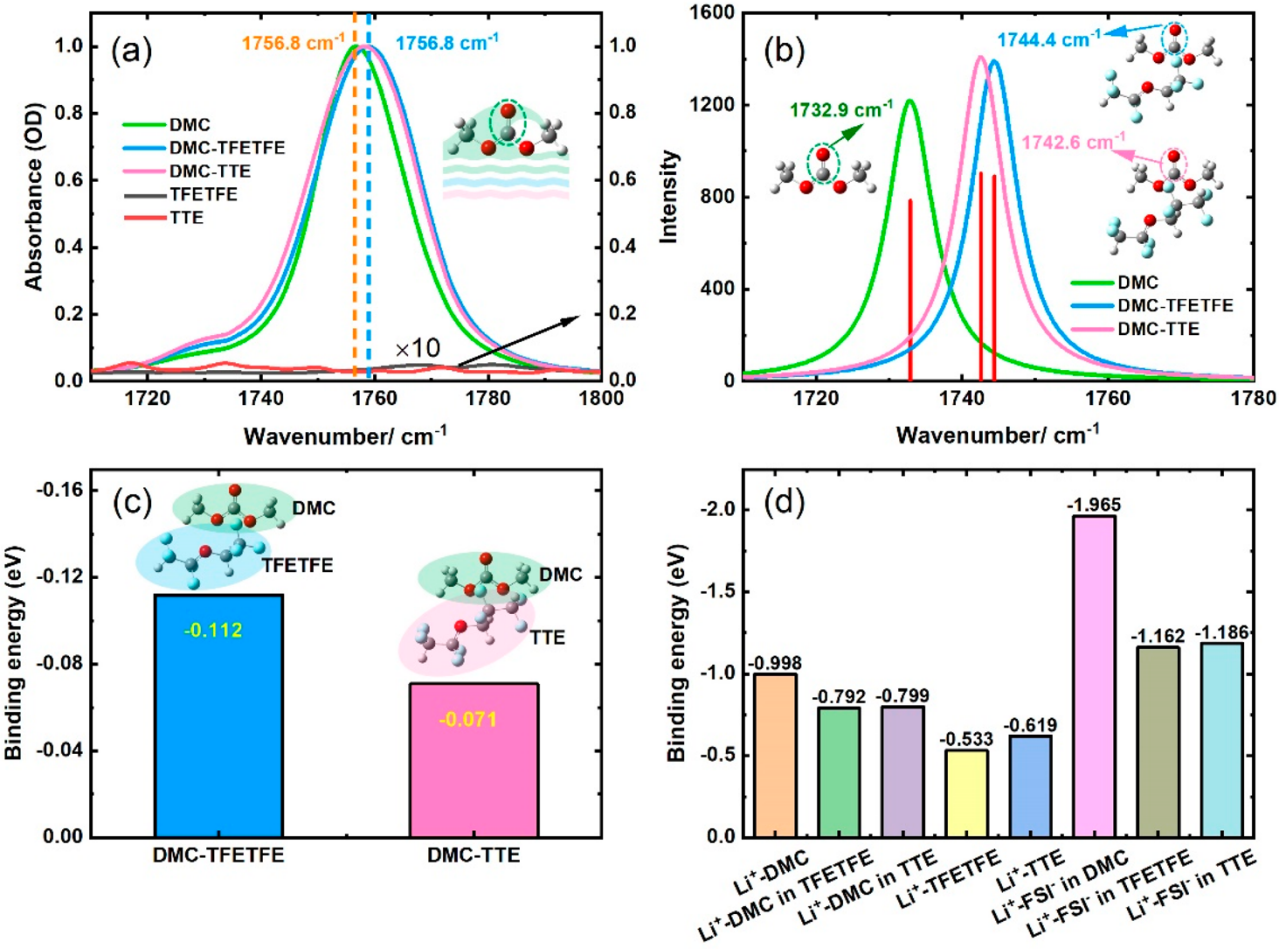
FTIR spectra of pure DMC, a DMC–antisolvent mixture, and antisolvents. (a) Experimental data and (b) DFT calculated FTIR spectra; the insets show the optimized geometric configurations. (c) The binding energy of DMC–antisolvent complexes; the colored structures represent the modes of interaction between DMC and antisolvents. (d) The binding energies between Li^+^ and various species are calculated by DFT.

To better understand the effects of antisolvents on solvation structure, the DMC/antisolvent mixed solutions are characterized using FTIR spectroscopy. As shown in Figure 2a, the vibration frequency of the C=O band of pure DMC is observed at 1756.8 cm^–1^, while it undergoes a blueshift to ∼1759 cm^–1^ and broadens with the addition of antisolvents. The frequency difference means that the antisolvent changes the charge distribution on the C=O group. Moreover, the FTIR spectra of antisolvents are also provided in Figure 2a. It shows that the FTIR spectra of TFETFE and TTE have almost no absorption at 10× magnification in the region around 1710–1800 cm^–1^. These results indicate that the antisolvents do not interfere with the accuracy of the FTIR results.

### 3.2 Solvation Structure Analysis Using DFT

To pinpoint the DMC/antisolvent complex structures, we utilize the DFT method to achieve structure optimizations and vibrational analyses of complexes. The calculated FTIR spectra are plotted in Figure 2b, which shows the same trend as experimental FTIR spectra. The optimized geometric configurations presented in Figure 2b are used to determine how the DMC molecules and antisolvents bond. These results indicate that the antisolvent electrostatic interaction with DMC from one side of the ester ether oxygen atom (O–C–O) results in an intensified inductive effect on the C=O of DMC, further causing a strong blueshift of C=O frequency. Conversely, this result will cause a reduction in the negative charge on the oxygen atom and concomitantly weaken the interaction between the C=O of DMC with Li^+^.

In addition, the binding energies of DMC-TFETFE and DMC-TTE in vacuum are determined to be −0.112 and −0.071 eV (Figure 2c), respectively. This means that the weaker interaction between DMC and TTE has less effect on the C=O···Li^+^, allowing DMC to be involved in the solvation shell. Moreover, the binding energy between Li^+^ and various species is calculated by DFT, as shown in Figure 2(d). The binding energy of Li^+^–antisolvent complexes (−0.533 and −0.619 eV) is found to be much weaker than that of Li^+^–DMC, which is consistent with the insolubility of antisolvent and agrees with previous reports.^30,52^ The dielectric constant (ε) can be an indicator of the polarity of a molecule. TFETFE (ε = 6.5) and TTE (ε = 6.2) are low-polarity diluents, whose dielectric constants are higher than that of DMC (ε = 3.09). The results show that the binding energy of the Li^+^–DMC complex decreases from −0.998 to approximately −0.79 eV, while the binding energy of Li^+^–FSI^–^ complex decreases from −1.965 to −1.16 eV with the addition of antisolvents. Noticeably, the binding energy of the Li^+^–FSI^–^ complex is decreased more than that of Li^+^–DMC complex at the antisolvent condition, indicating that antisolvents would prevent the association of FSI^–^ and Li^+^. In other words, the weakened Li^+^–FSI^–^ association is more likely to promote participation of DMC solvents in the Li^+^ solvation sheath, which is also consistent with the FTIR spectra. For desolvation energy, it is important for the desolvation process, and the weaker ion desolvation energy is beneficial for ion diffusion between the electrolyte/electrode interface and improves the rate performance of batteries.^53−56^ In addition, the positive correlation between desolvation energy and binding energy has been demonstrated by many works.^57−60^ Therefore, the reduced binding energy of the Li^+^···DMC and Li^+^···FSI^–^ interactions reveals that the Li^+^ desolvation energy of the solvated structure is lower in LHCEs. Hence, the LHCEs can effectively improve the rate performance of LIBs.

### 3.3 Solvation Structure Analysis by MD Calculation

**Figure 3:**
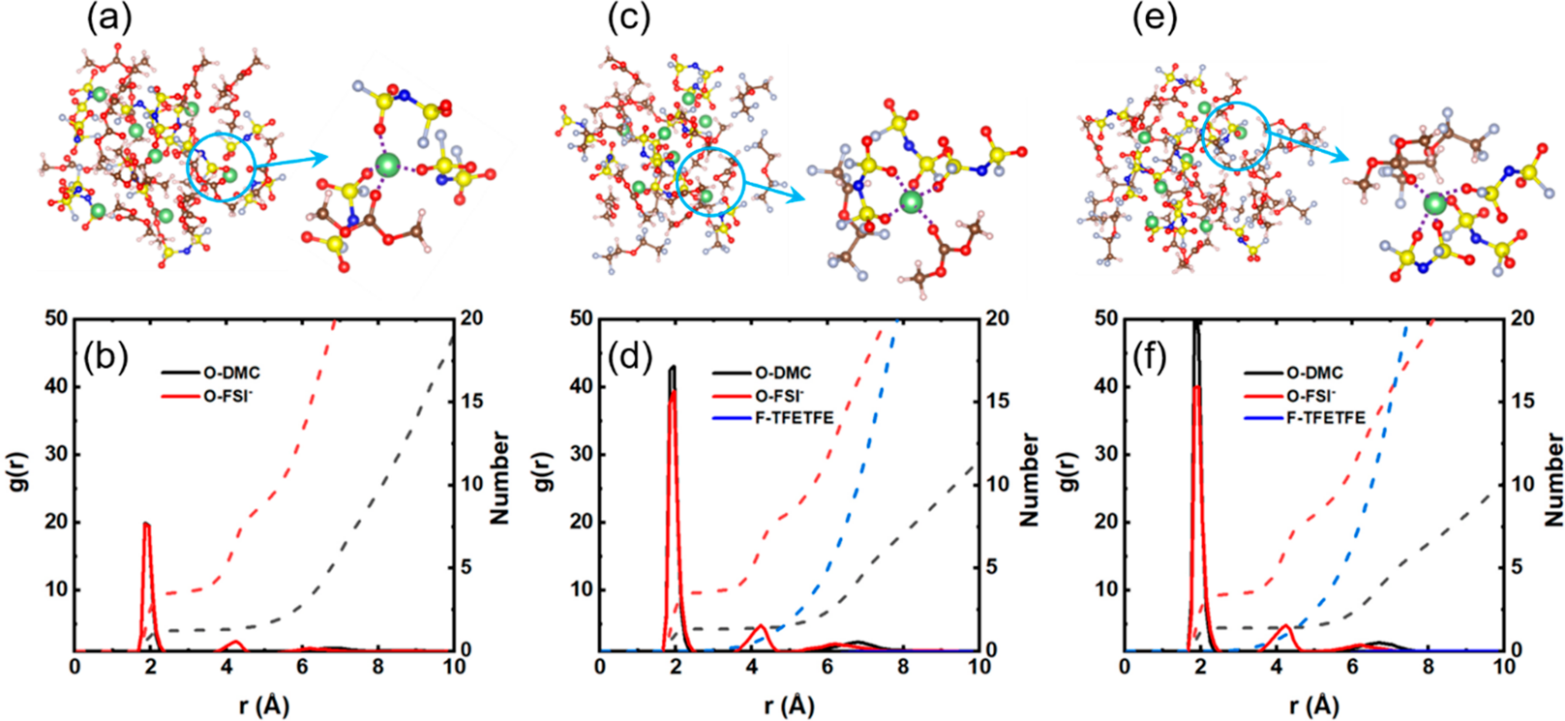
Li-ion solvation environment along with the first solvation shells (a,c,e) and the corresponding RDFs along with coordination numbers (b,d,f) in the LiFSI electrolyte. Structural information about the Li^+^ in the HCE (a,b), LHCE–TFETFE (c,d), and LHCE–TTE (e,f). Color code of the spheres: green: Li, red: O, silver: F, blue: N, pink: H, yellow: S, and brown: C.

MD simulations are further conducted to corroborate the solvation structure. Figure 3 shows snapshots of the simulated electrolyte structure along with the radial distribution function (RDF, solid lines) and coordination numbers (dashed lines). The MD results are tabulated (Table 2) and show that the FSI^–^ anion has a higher coordination number with Li^+^ than DMC molecules. The minimum (*r*_*min*_ = 3.05 Å) of the first peak is chosen as the cutoff distance to count the coordination number of DMC and FSI^–^ anions. The position of the first peak corresponding to Li^+^–O_DMC_ and Li^+^–O_FSI_^–^ was centered at about 1.85 Å, and each Li^+^ is solvated by 1.25 DMC and 3.63 FSI^–^ in the HCE. For the LHCE–TFETFE and LHCE–TTE electrolytes, the coordination numbers of DMC increase to 1.34 and 1.40, and the coordination numbers of FSI^–^ anion are 3.63 and 3.49 in the Li^+^ solvation structure, agreeing with the previous FTIR spectra and DFT analyses. For LHCE–TFETFE, a peak of the Li^+^–O_DMC_ RDF is present at 1.95 Å, while it is located at 1.85 Å in LHCE–TTE, which further confirms that the different antisolvents will change the solvation structure.

**Table 2:** Effect of Antisolvent on the Maxima (*r*_*max*_) and Minima (*r*_*min*_) of the First Peak, Radial Distribution Functions *g*(*r*_*max*_), and the Coordination Number *N*(*r*_*min*_) of Different Electrolytes

Pair	System	*T*/K	*r*_*max*_/Å	*g*(*r*_*max*_)	*R*_*min*_/Å	*N*(*r*_*min*_)
Li^+^–O_DMC_	4.5 M	298	1.85	19.92	3.25	1.254
	LHCE–TFETFE		1.95	43.13	3.25	1.342
	LHCE–TTE		1.85	51.99	3.25	1.397
Li^+^–O_FSI_^–^	4.5 M	298	1.85	19.76	3.05	3.632
	LHCE–TFETFE		1.95	39.44	3.05	3.625
	LHCE–TTE		1.95	40.10	3.05	3.494

**Figure 4:**
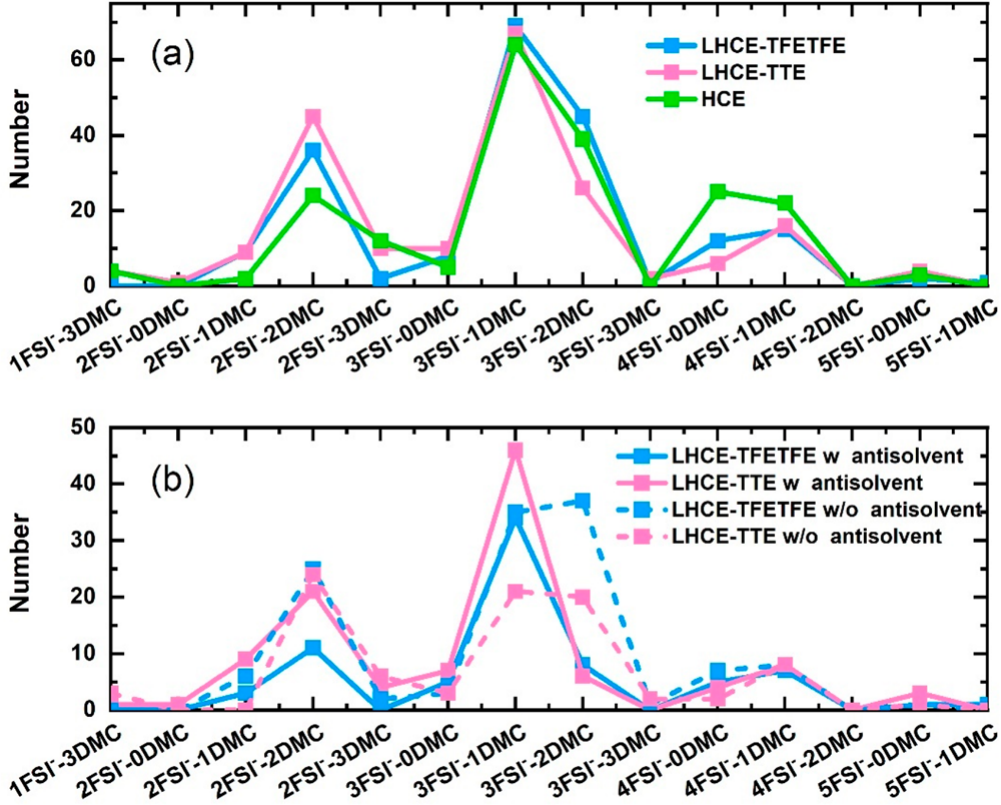
Population of Li^+^ solvation structures in HCE and LHCEs extracted from MD simulation. (a) The population of the total Li^+^ solvation structures in three kinds of electrolytes. (b) The population of the Li^+^ solvation structures with (solid line) or without (dashed line) connection to antisolvent in LHCEs.

**Figure 5:**
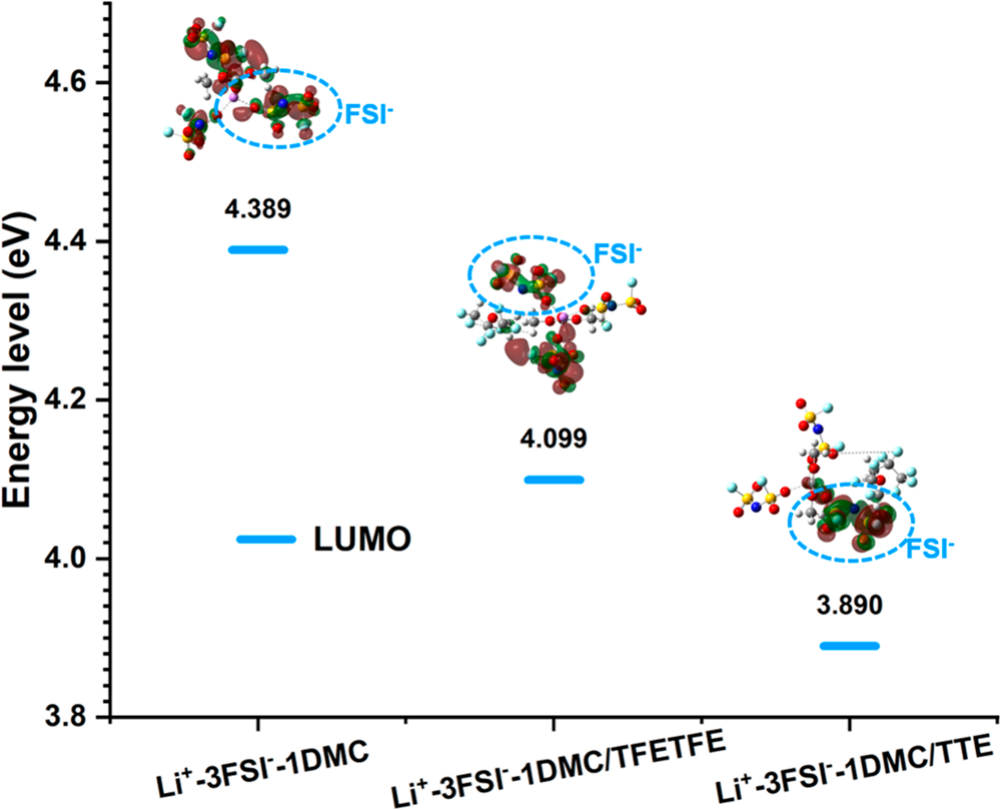
Frontier molecular orbital energy of the Li^+^ solvation sheath in HCE and LHCEs obtained by DFT calculations. The insets show the molecular orbital states of the typical Li^+^ coordination structure.

**Figure 6:**
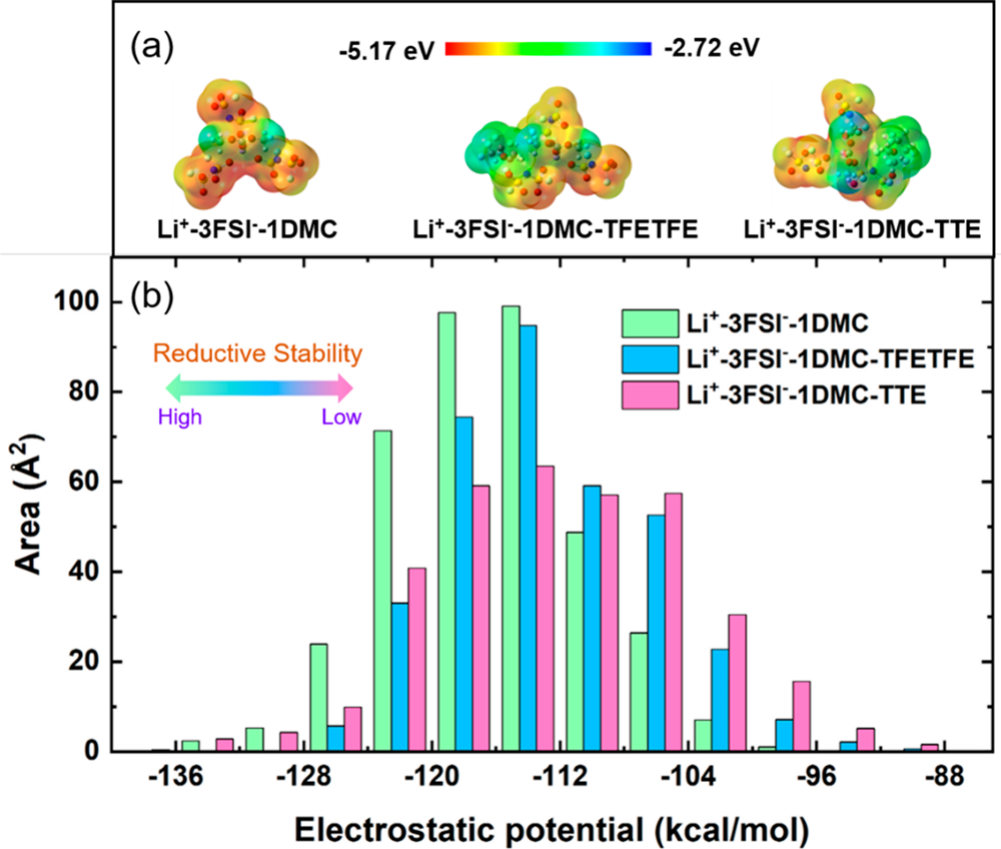
(a) Electrostatic potential mapping of Li^+^ solvation structures and (b) the surface area in each ESP range on the vdW surface of the FSI^–^ anion part.

According to the statistics of MD simulations, the percentage of Li ion coordination environments in different electrolytes is displayed in Figure 4a: (1) Li^+^ coordinates with 2FSI^–^ and 2DMC (Li^+^–2FSI^–^–2DMC), (2) Li^+^ coordinates with 3FSI^–^ and 1DMC (Li^+^–3FSI^–^–DMC), and (3) Li^+^ coordinates with 3FSI^–^ and 2DMC (Li^+^–3FSI^–^–2DMC) clusters. Noticeably, the antisolvents cause the solvated clusters of high anion coordination components to decrease, while the solvated cluster of the low anion coordination increases. As shown in Figure 4b, the dominant coordination structures of Li^+^ connect with antisolvents are Li^+^–2FSI^–^–2DMC and Li^+^–3FSI^–^–DMC in LHCEs. To evaluate the influence of the antisolvents around the cluster on the solvation structure energy level, frontier molecular orbital analysis was conducted by means of DFT calculations. It can be seen from Figure 5 that antisolvents have a significant effect on the lowest unoccupied molecular orbital (LUMO) energy level of the Li^+^ solvated cluster: the LUMO energy is more negative for Li^+^–3FSI^–^–1DMC. The lower LUMO energy reflects the decreased reduction stability of solvated clusters. In the subgraph, LUMO is localized on the FSI^–^ anions, suggesting that FSI^–^ anions are the primary active sites of reduction and that a lower LUMO energy increases the possibility of FSI^–^ decomposition. Moreover, the electrostatic potential (ESP) mapping in Figure 6a also demonstrates that the distribution of negative charges on the surface of solvation clusters is weakened due to the presence of antisolvents. The surface area in different ESP ranges (Figure 6b) allows us to quantitatively analyze the characteristics of the molecular surface charge. The reduced relative abundance of ESP distribution on FSI^–^ anions suggests that the reduction stability of FSI^–^ is reduced in the presence of antisolvents. In previous reports, the LHCE–TTE electrolytes demonstrate better cycling performance of Li||Cu cells (98.9 and 99.6%) than LHCE–TFETFE (98.2 and 99.4%).^30^ Therefore, these experimental results prove that the preferential decomposition of FSI^–^ anions facilitates the formation of a stable anion-derived solid-electrolyte interphase (SEI).^30,61,62^

**Figure 7:**
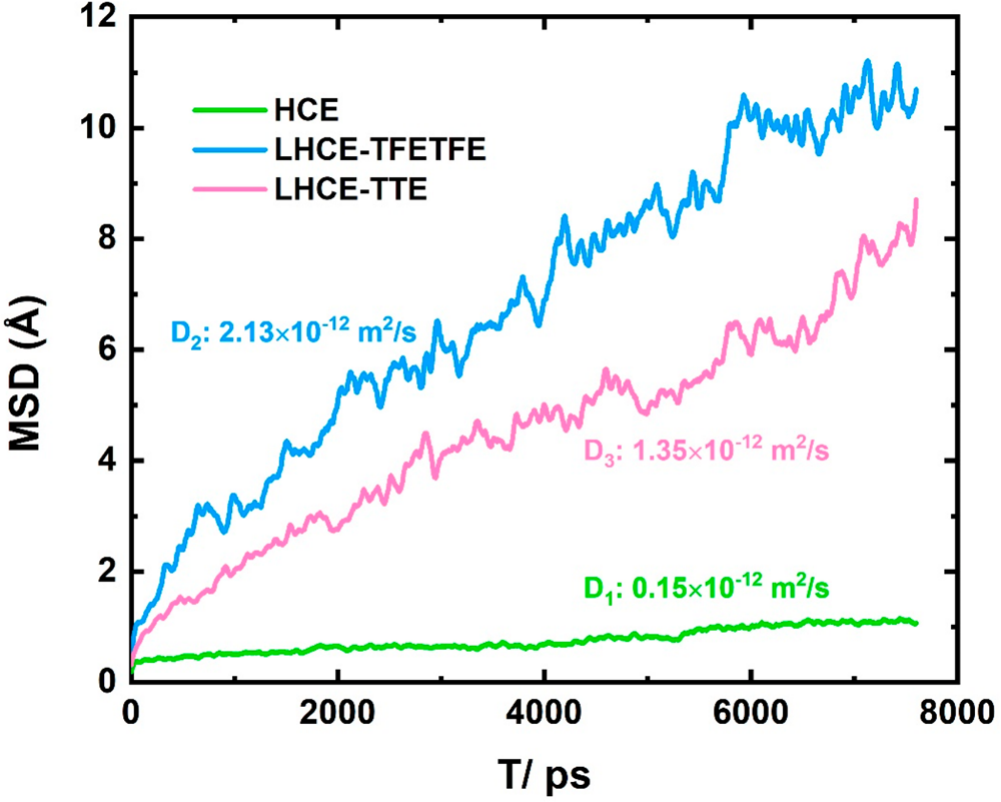
Mean square displacement (MSD) of Li^+^ at two kinds of LHCEs.

Furthermore, to assess the influence of antisolvent on the transport properties of ions, we extract the diffusion coefficient (*D*) of Li^+^ from the trajectory of the MD simulations based on eq 3
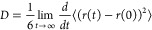
3where *r(t)* is the location of Li^+^ at time *t* and ⟨⟩ represents an ensemble average. As shown in Figure 7, the MSD of Li^+^ vs time shows the increased displacement of Li^+^ with the addition of antisolvents. According to eq 3, the diffusion coefficient of Li^+^ (*D*_*Li*_) in HCE is 0.15 × 10^–12^ m^2^/s. Notably, in LHCEs, the diffusion coefficient is enhanced by 10 times (2.13 × 10^–12^ m^2^/s for LHCE–TFETFE and 1.35 × 10^–12^ m^2^/s for LHCE–TTE). The enhanced transport mechanisms manifest that antisolvents can facilitate Li-ion transport, and the amount of increase depends on the type of antisolvent. In HCEs, structural motion is the dominant mode, which denotes ion diffusion through the exchange of ion association/dissociation between different solvation structures.^63,64^ The microstate of LHCEs is similar to that of HCE, so the lower binding energy of the Li^+^···DMC and Li^+^···FSI^–^ interactions is conducive to Li^+^ overcoming the migration barrier. In addition, the higher Li^+^ transport can attenuate the ion concentration gradient and reduce the effect of concentration polarization, which is favorable to obtain excellent electrochemical performance of the battery.^11^

## 4 Conclusions

In this work, we employ FTIR spectra, DFT, and MD simulations to examine the structural and dynamic properties of LHCEs and discover the inescapable effect of antisolvent on the solvation structure, energy level, and transport properties of LHCEs. More specifically, antisolvents provide a lower dielectric environment, leading to increased participation of DMC molecules and decreased participation of FSI^–^ anions in the Li^+^ solvation sheath. However, the intensified inductive effect caused by antisolvents reduces the binding energy of the Li^+^···DMC interactions and changes with various antisolvents. The binding energies of the Li^+^···DMC and Li^+^···FSI^–^ interactions are decreased with the addition of antisolvent compared with the superconcentrated electrolytes, suggesting that the antisolvents could help to lower the desolvation energy and facilitate interface kinetics in the electrochemical reaction. Furthermore, antisolvents in the second shell change the charge distribution on the surface of the solvation cluster and reduce the reduction stability of FSI^–^. Therefore, the promoted anion-derived solid-electrolyte interphase (SEI) in LHCEs is found to agree with the experimental results. In addition, the MD results show that the addition of antisolvents can enhance Li^+^ transport. This study elucidates the contribution of antisolvent regulate interfacial chemistry in LHCEs, and we hope our work can provide guidance for advanced LHCEs for high-performance batteries.

## Supporting Information Available

Transparent Peer Review report available (PDF)

## References

[ref1] Goodenough J. B.; Kim Y. Challenges for Rechargeable Li Batteries. Chem. Mater. 2010, 22, 587–603. 10.1021/cm901452z.

[ref2] Tarascon J. M.; Armand M. Issues and Challenges Facing Rechargeable Lithium Batteries. Nature 2001, 414, 359–367. 10.1038/35104644. 11713543

[ref3] Qian J.; Henderson W. A.; Xu W.; Bhattacharya P.; Engelhard M.; Borodin O.; Zhang J. G. High Rate and Stable Cycling of Lithium Metal Anode. Nat. Commun. 2015, 6, 6362 10.1038/ncomms7362. 25698340 PMC4346622

[ref4] Piao N.; Liu S.; Zhang B.; Ji X.; Fan X.; Wang L.; Wang P.-F.; Jin T.; Liou S.-C.; Yang H.; Jiang J.; Xu K.; Schroeder M. A.; He X.; Wang C. Lithium Metal Batteries Enabled by Synergetic Additives in Commercial Carbonate Electrolytes. ACS Energy Lett. 2021, 6, 1839–1848. 10.1021/acsenergylett.1c00365.

[ref5] Kim K.; Ma H.; Park S.; Choi N.-S. Electrolyte-Additive-Driven Interfacial Engineering for High-Capacity Electrodes in Lithium-Ion Batteries: Promise and Challenges. ACS Energy Lett. 2020, 5, 1537–1553. 10.1021/acsenergylett.0c00468.

[ref6] Aupperle F.; von Aspern N.; Berghus D.; Weber F.; Eshetu G. G.; Winter M.; Figgemeier E. The Role of Electrolyte Additives on the Interfacial Chemistry and Thermal Reactivity of Si-Anode-Based Li-Ion Battery. ACS Appl. Energy Mater. 2019, 2, 6513–6527. 10.1021/acsaem.9b01094.

[ref7] Yamada Y.; Furukawa K.; Sodeyama K.; Kikuchi K.; Yaegashi M.; Tateyama Y.; Yamada A. Unusual Stability of Acetonitrile-Based Superconcentrated Electrolytes for Fast-Charging Lithium-Ion Batteries. J. Am. Chem. Soc. 2014, 136, 5039–5046. 10.1021/ja412807w. 24654781

[ref8] Yamada Y.; Wang J.; Ko S.; Watanabe E.; Yamada A. Advances and Issues in Developing Salt-Concentrated Battery Electrolytes. Nat. Energy 2019, 4, 269–280. 10.1038/s41560-019-0336-z.

[ref9] Chen S.; Zheng J.; Mei D.; Han K. S.; Engelhard M. H.; Zhao W.; Xu W.; Liu J.; Zhang J. G. High-Voltage Lithium-Metal Batteries Enabled by Localized High-Concentration Electrolytes. Adv. Mater. 2018, 30, 1706102 10.1002/adma.201706102. 29575163

[ref10] Ma G.; Wang L.; He X.; Zhang J.; Chen H.; Xu W.; Ding Y. Pseudoconcentrated Electrolyte with High Ionic Conductivity and Stability Enables High-Voltage Lithium-Ion Battery Chemistry. ACS Appl. Energy Mater. 2018, 1, 5446–5452. 10.1021/acsaem.8b01020.

[ref11] Piao N.; Ji X.; Xu H.; Fan X.; Chen L.; Liu S.; Garaga M. N.; Greenbaum S. G.; Wang L.; Wang C.; He X. Countersolvent Electrolytes for Lithium-Metal Batteries. Adv. Energy Mater. 2020, 10, 1903568 10.1002/aenm.201903568.

[ref12] Perez Beltran S.; Cao X.; Zhang J.-G.; Balbuena P. B. Localized High Concentration Electrolytes for High Voltage Lithium–Metal Batteries: Correlation between the Electrolyte Composition and Its Reductive/Oxidative Stability. Chem. Mater. 2020, 32, 5973–5984. 10.1021/acs.chemmater.0c00987.

[ref13] Lee S.; Park K.; Koo B.; Park C.; Jang M.; Lee H.; Lee H. Safe, Stable Cycling of Lithium Metal Batteries with Low-Viscosity, Fire-Retardant Locally Concentrated Ionic Liquid Electrolytes. Adv. Funct. Mater. 2020, 30, 2003132 10.1002/adfm.202003132.

[ref14] Takada K.; Yamada Y.; Yamada A. Optimized Nonflammable Concentrated Electrolytes by Introducing a Low-Dielectric Diluent. ACS Appl. Mater. Interfaces 2019, 11, 35770–35776. 10.1021/acsami.9b12709. 31498585

[ref15] Ren X.; Zhang X.; Shadike Z.; Zou L.; Jia H.; Cao X.; Engelhard M. H.; Matthews B. E.; Wang C.; Arey B. W.; Yang X. Q.; Liu J.; Zhang J. G.; Xu W. Designing Advanced in Situ Electrode/Electrolyte Interphases for Wide Temperature Operation of 4.5 V Li||LiCoO_2_ Batteries. Adv. Mater. 2020, 32, 2004898 10.1002/adma.202004898. 33150628

[ref16] Ren X.; Gao P.; Zou L.; Jiao S.; Cao X.; Zhang X.; Jia H.; Engelhard M. H.; Matthews B. E.; Wu H.; Lee H.; Niu C.; Wang C.; Arey B. W.; Xiao J.; Liu J.; Zhang J. G.; Xu W. Role of Inner Solvation Sheath within Salt-Solvent Complexes in Tailoring Electrode/Electrolyte Interphases for Lithium Metal Batteries. Proc. Natl. Acad. Sci. U.S.A. 2020, 117, 28603–28613. 10.1073/pnas.2010852117. 33144505 PMC7682554

[ref17] Zhang X.; Jia H.; Zou L.; Xu Y.; Mu L.; Yang Z.; Engelhard M. H.; Kim J.-M.; Hu J.; Matthews B. E.; Niu C.; Wang C.; Xin H.; Lin F.; Xu W. Electrolyte Regulating toward Stabilization of Cobalt-Free Ultrahigh-Nickel Layered Oxide Cathode in Lithium-Ion Batteries. ACS Energy Lett. 2021, 6, 1324–1332. 10.1021/acsenergylett.1c00374.

[ref18] Ren X.; Chen S.; Lee H.; Mei D.; Engelhard M. H.; Burton S. D.; Zhao W.; Zheng J.; Li Q.; Ding M. S.; Schroeder M.; Alvarado J.; Xu K.; Meng Y. S.; Liu J.; Zhang J.-G.; Xu W. Localized High-Concentration Sulfone Electrolytes for High-Efficiency Lithium-Metal Batteries. Chem. 2018, 4, 1877–1892. 10.1016/j.chempr.2018.05.002.

[ref19] Lin S.; Hua H.; Li Z.; Zhao J. Functional Localized High-Concentration Ether-Based Electrolyte for Stabilizing High-Voltage Lithium-Metal Battery. ACS Appl. Mater. Interfaces 2020, 12, 33710–33718. 10.1021/acsami.0c07904. 32597632

[ref20] Alfonso-Hernandez L.; Oldani N.; Athanasopoulos S.; Lupton J. M.; Tretiak S.; Fernandez-Alberti S. Photoinduced Energy Transfer in Linear Guest-Host Chromophores: A Computational Study. J. Phys. Chem. A 2021, 125, 5303–5313. 10.1021/acs.jpca.1c02644. 34106721

[ref21] Cao X.; Ren X.; Zou L.; Engelhard M. H.; Huang W.; Wang H.; Matthews B. E.; Lee H.; Niu C.; Arey B. W.; Cui Y.; Wang C.; Xiao J.; Liu J.; Xu W.; Zhang J.-G. Monolithic Solid–Electrolyte Interphases Formed in Fluorinated Orthoformate-Based Electrolytes Minimize Li Depletion and Pulverization. Nat. Energy 2019, 4, 796–805. 10.1038/s41560-019-0464-5.

[ref22] Yu L.; Chen S.; Lee H.; Zhang L.; Engelhard M. H.; Li Q.; Jiao S.; Liu J.; Xu W.; Zhang J.-G. A Localized High-Concentration Electrolyte with Optimized Solvents and Lithium Difluoro(Oxalate)Borate Additive for Stable Lithium Metal Batteries. ACS Energy Lett. 2018, 3, 2059–2067. 10.1021/acsenergylett.8b00935.

[ref23] Shin W.; Zhu L.; Jiang H.; Stickle W. F.; Fang C.; Liu C.; Lu J.; Ji X. Fluorinated Co-Solvent Promises Li-S Batteries under Lean-Electrolyte Conditions. Mater. Today 2020, 40, 63–71. 10.1016/j.mattod.2020.06.007.

[ref24] Shin M.; Wu H. L.; Narayanan B.; See K. A.; Assary R. S.; Zhu L.; Haasch R. T.; Zhang S.; Zhang Z.; Curtiss L. A.; Gewirth A. A. Effect of the Hydrofluoroether Cosolvent Structure in Acetonitrile-Based Solvate Electrolytes on the Li^+^ Solvation Structure and Li-S Battery Performance. ACS Appl. Mater. Interfaces 2017, 9, 39357–39370. 10.1021/acsami.7b11566. 29045124

[ref25] Dong X.; Lin Y.; Li P.; Ma Y.; Huang J.; Bin D.; Wang Y.; Qi Y.; Xia Y. High-Energy Rechargeable Metallic Lithium Battery at – 70 °C Enabled by a Cosolvent Electrolyte. Angew. Chem., Int. Ed. 2019, 131, 5679–5683. 10.1002/ange.201900266. 30821403

[ref26] Lin S.; Hua H.; Lai P.; Zhao J. A Multifunctional Dual-Salt Localized High-Concentration Electrolyte for Fast Dynamic High-Voltage Lithium Battery in Wide Temperature Range. Adv. Energy Mater. 2021, 11, 2101775 10.1002/aenm.202101775.

[ref27] Chen S.; Zheng J.; Yu L.; Ren X.; Engelhard M. H.; Niu C.; Lee H.; Xu W.; Xiao J.; Liu J.; Zhang J.-G. High-Efficiency Lithium Metal Batteries with Fire-Retardant Electrolytes. Joule 2018, 2, 1548–1558. 10.1016/j.joule.2018.05.002.

[ref28] Cao X.; Xu Y.; Zhang L.; Engelhard M. H.; Zhong L.; Ren X.; Jia H.; Liu B.; Niu C.; Matthews B. E.; Wu H.; Arey B. W.; Wang C.; Zhang J.-G.; Xu W. Nonflammable Electrolytes for Lithium Ion Batteries Enabled by Ultraconformal Passivation Interphases. ACS Energy Lett. 2019, 4, 2529–2534. 10.1021/acsenergylett.9b01926.

[ref29] Cao X.; Gao P.; Ren X.; Zou L.; Engelhard M. H.; Matthews B. E.; Hu J.; Niu C.; Liu D.; Arey B. W.; Wang C.; Xiao J.; Liu J.; Xu W.; Zhang J. G. Effects of Fluorinated Solvents on Electrolyte Solvation Structures and Electrode/Electrolyte Interphases for Lithium Metal Batteries. Proc. Natl. Acad. Sci. U.S.A. 2021, 118, e2020357118 10.1073/pnas.2020357118. 33632763 PMC7936379

[ref30] Ding J. F.; Xu R.; Yao N.; Chen X.; Xiao Y.; Yao Y. X.; Yan C.; Xie J.; Huang J. Q. Non-Solvating and Low-Dielectricity Cosolvent for Anion-Derived Solid Electrolyte Interphases in Lithium Metal Batteries. Angew.Chem. Int. Ed. 2021, 60, 11442–11447. 10.1002/anie.202101627. 33655631

[ref31] Cao X.; Jia H.; Xu W.; Zhang J.-G. Review—Localized High-Concentration Electrolytes for Lithium Batteries. J. Electrochem. Soc. 2021, 168, 010522 10.1149/1945-7111/abd60e.

[ref32] Zhou X.; Zhang Q.; Zhu Z.; Cai Y.; Li H.; Li F. Anion-Reinforced Solvation for a Gradient Inorganic-Rich Interphase Enables High-Rate and Stable Sodium Batteries. Angew.Chem. Int. Ed. 2022, 61, e202205045 10.1002/anie.202205045. 35533111

[ref33] Chen X.; Qin L.; Sun J.; Zhang S.; Xiao D.; Wu Y. Phase Transfer-Mediated Degradation of Ether-Based Localized High-Concentration Electrolytes in Alkali Metal Batteries. Angew. Chem. Int. Ed. 2022, 61, e202207018 10.1002/anie.202207018. PMC9541886 35695829

[ref34] Chen X.; Zhang X.-Q.; Li H.-R.; Zhang Q. Cation–Solvent, Cation–Anion, and Solvent–Solvent Interactions with Electrolyte Solvation in Lithium Batteries. Batteries Supercaps 2019, 2, 128–131. 10.1002/batt.201800118.

[ref35] Xing L.; Li W.; Wang C.; Gu F.; Xu M.; Tan C.; Yi J. Theoretical Investigations on Oxidative Stability of Solvents and Oxidative Decomposition Mechanism of Ethylene Carbonate for Lithium Ion Battery Use. J. Phys. Chem. B 2009, 113, 16596–16602. 10.1021/jp9074064. 19947609

[ref36] Marenich A. V.; Cramer C. J.; Truhlar D. G. Universal Solvation Model Based on Solute Electron Density and on a Continuum Model of the Solvent Defined by the Bulk Dielectric Constant and Atomic Surface Tensions. J. Phys. Chem. B 2009, 113, 6378–6396. 10.1021/jp810292n. 19366259

[ref37] Frisch M. J.; Trucks G. W.; Schlegel H. B.; Scuseria G. E.; Robb M. A.; Cheeseman J. R.; Scalmani G.; Barone V.; Petersson G. A.; Nakatsuji H.; Li X.; Caricato M.; Marenich A. V.; Blolino J.; Janesko B. G.; Gomperts R.; Mennucci B.; Hratchian H. P.; Ortiz J. V.; Izmaylov A. F.; Sonnenberg J. L.; Williams-Young D.; Ding F.; Lipparini F.; Egidi F.; Goings J.; Peng B.; Petrone A.; Henderson T.; Ranasinghe D.; Zakrzewski V. G.; Gao J.; Rega N.; Zheng G.; Liang W.; Hada M.; Ehara M.; Toyota K.; Fukuda R.; Hasegawa J.; Ishida M.; Nakajima T.; Honda Y.; Kitao O.; Nakai H.; Vreven T.; Throssell K.; Montgomery J. A. Jr.; Peralta J. E.; Oligaro F.; Bearpark M. J.; Heyd J. J; Brothers E. N.; Kudin K. N.; Staroverov V. N.; Keith T. A.; Kobayashi R.; Normand J.; Ragavachari K.; Rendell A. P.; Burant J. C.; Iyengar S. S.; Tomasi J.; Cossi M.; Millam J. M.; Klene M.; Adamo C.; Cammi R.; Ochterski J. W.; Martin R. L.; Morokuma K.; Farkas O.; Foresman J. B.; Fox D. J. Gaussian 16, revision C.01; Gaussian, Inc.: Wallingford, CT, 2016.

[ref38] Lu T.; Chen F. Multiwfn: A Multifunctional Wavefunction Analyzer. J. Comput. Chem. 2012, 33, 580–592. 10.1002/jcc.22885. 22162017

[ref39] Plimpton S. Fast Parallel Algorithms for Short-Range Molecular Dynamics. J. Comput. Phys. 1995, 117, 1–19. 10.1006/jcph.1995.1039.

[ref40] Kumar N.; Seminario J. M. Lithium-Ion Model Behavior in an Ethylene Carbonate Electrolyte Using Molecular Dynamics. J. Phys. Chem. C 2016, 120, 16322–16332. 10.1021/acs.jpcc.6b03709.

[ref41] Mallarapu A.; Bharadwaj V. S.; Santhanagopalan S. Understanding Extreme Fast Charge Limitations in Carbonate Mixtures. J. Mater. Chem. A 2021, 9, 4858–4869. 10.1039/D0TA10166D.

[ref42] Jakalian A.; Jack D. B.; Bayly C. I. Fast, Efficient Generation of High-Quality Atomic Charges. Am1-Bcc Model: Ii. Parameterization and Validation. J. Comput. Chem. 2002, 23, 1623–1641. 10.1002/jcc.10128. 12395429

[ref43] Wang J.; Wolf R. M.; Caldwell J. W.; Kollman P. A.; Case D. A. Development and Testing of a General Amber Force Field. J. Comput. Chem. 2004, 25, 1157–1174. 10.1002/jcc.20035. 15116359

[ref44] Canongia Lopes J. N.; Pádua A. A. H. Molecular Force Field for Ionic Liquids Composed of Triflate or Bistriflylimide Anions. J. Phys. Chem. B 2004, 108, 16893–16898. 10.1021/jp0476545.

[ref45] Shimizu K.; Almantariotis D.; Costa Gomes M. F.; Padua A. A.; Canongia Lopes J. N. Molecular Force Field for Ionic Liquids V: Hydroxyethylimidazolium, Dimethoxy-2- Methylimidazolium, and Fluoroalkylimidazolium Cations and Bis(Fluorosulfonyl)Amide, Perfluoroalkanesulfonylamide, and Fluoroalkylfluorophosphate Anions. J. Phys. Chem. B 2010, 114, 3592–3600. 10.1021/jp9120468. 20175555

[ref46] Martinez L.; Andrade R.; Birgin E. G.; Martinez J. M. Packmol: A Package for Building Initial Configurations for Molecular Dynamics Simulations. J. Comput. Chem. 2009, 30, 2157–2164. 10.1002/jcc.21224. 19229944

[ref47] Jewett A. I.; Stelter D.; Lambert J.; Saladi S. M.; Roscioni O. M.; Ricci M.; Autin L.; Maritan M.; Bashusqeh S. M.; Keyes T.; Dame R. T.; Shea J. E.; Jensen G. J.; Goodsell D. S. Moltemplate: A Tool for Coarse-Grained Modeling of Complex Biological Matter and Soft Condensed Matter Physics. J. Mol. Biol. 2021, 433, 166841 10.1016/j.jmb.2021.166841. 33539886 PMC8119336

[ref48] Humphrey W.; Dalke A.; Schulten K. Vmd: Visual Molecular Dynamics. J. Mol. Graphics 1996, 14, 33–38. 10.1016/0263-7855(96)00018-5. 8744570

[ref49] Momma K.; Izumi F. Vesta 3for Three-Dimensional Visualization of Crystal, Volumetric and Morphology Data. J. Appl. Crystallogr. 2011, 44, 1272–1276. 10.1107/S0021889811038970.

[ref50] Seo D. M.; Reininger S.; Kutcher M.; Redmond K.; Euler W. B.; Lucht B. L. Role of Mixed Solvation and Ion Pairing in the Solution Structure of Lithium Ion Battery Electrolytes. J. Phys. Chem. C 2015, 119, 14038–14046. 10.1021/acs.jpcc.5b03694.

[ref51] Nie M.; Abraham D. P.; Seo D. M.; Chen Y.; Bose A.; Lucht B. L. Role of Solution Structure in Solid Electrolyte Interphase Formation on Graphite with Lipf6 in Propylene Carbonate. J. Phys. Chem. C 2013, 117, 25381–25389. 10.1021/jp409765w.

[ref52] Zhang X.; Zou L.; Xu Y.; Cao X.; Engelhard M. H.; Matthews B. E.; Zhong L.; Wu H.; Jia H.; Ren X.; Gao P.; Chen Z.; Qin Y.; Kompella C.; Arey B. W.; Li J.; Wang D.; Wang C.; Zhang J. G.; Xu W. Advanced Electrolytes for Fast-Charging High-Voltage Lithium-Ion Batteries in Wide-Temperature Range. Adv. Energy Mater. 2020, 10, 2000368 10.1002/aenm.202000368.

[ref53] Tang Z.; Wang H.; Wu P. F.; Zhou S. Y.; Huang Y. C.; Zhang R.; Sun D.; Tang Y. G.; Wang H. Y. Electrode-Electrolyte Interfacial Chemistry Modulation for Ultra-High Rate Sodium-Ion Batteries. Angew.Chem. Int. Ed. 2022, 61, e202200475 10.1002/anie.202200475. 35199431

[ref54] Yao N.; Chen X.; Fu Z. H.; Zhang Q. Applying Classical, Ab Initio, and Machine-Learning Molecular Dynamics Simulations to the Liquid Electrolyte for Rechargeable Batteries. Chem. Rev. 2022, 122, 10970 10.1021/acs.chemrev.1c00904. 35576674

[ref55] Zhang N.; Deng T.; Zhang S.; Wang C.; Chen L.; Wang C.; Fan X. Critical Review on Low-Temperature Li-Ion/Metal Batteries. Adv. Mater. 2022, 34, 2107899 10.1002/adma.202107899. 34855260

[ref56] Li Q.; Liu G.; Cheng H.; Sun Q.; Zhang J.; Ming J. Low-Temperature Electrolyte Design for Lithium-Ion Batteries: Prospect and Challenges. Chemistry 2021, 27, 15842–15865. 10.1002/chem.202101407. 34558737

[ref57] Nan B.; Chen L.; Rodrigo N. D.; Borodin O.; Piao N.; Xia J.; Pollard T.; Hou S.; Zhang J.; Ji X.; Xu J.; Zhang X.; Ma L.; He X.; Liu S.; Wan H.; Hu E.; Zhang W.; Xu K.; Yang X. Q.; Lucht B.; Wang C. Enhancing Li(+) Transport in Nmc811||Graphite Lithium-Ion Batteries at Low Temperatures by Using Low-Polarity-Solvent Electrolytes. Angew. Chem. Int. Ed. 2022, 61, e202205967 10.1002/anie.202205967. 35789166

[ref58] Liang H. J.; Gu Z. Y.; Zhao X. X.; Guo J. Z.; Yang J. L.; Li W. H.; Li B.; Liu Z. M.; Li W. L.; Wu X. L. Ether-Based Electrolyte Chemistry Towards High-Voltage and Long-Life Na-Ion Full Batteries. Angew.Chem. Int. Ed. 2021, 60, 26837–26846. 10.1002/anie.202112550. 34636126

[ref59] Holoubek J.; Liu H.; Wu Z.; Yin Y.; Xing X.; Cai G.; Yu S.; Zhou H.; Pascal T. A.; Chen Z.; Liu P. Tailoring Electrolyte Solvation for Li Metal Batteries Cycled at Ultra-Low Temperature. Nat. Energy 2021, 6, 303 10.1038/s41560-021-00783-z. PMC7954221 33717504

[ref60] Okoshi M.; Yamada Y.; Yamada A.; Nakai H. Theoretical Analysis on De-Solvation of Lithium, Sodium, and Magnesium Cations to Organic Electrolyte Solvents. J. Electrochem. Soc. 2013, 160, A2160–A2165. 10.1149/2.074311jes.

[ref61] Li T.; Zhang X. Q.; Yao N.; Yao Y. X.; Hou L. P.; Chen X.; Zhou M. Y.; Huang J. Q.; Zhang Q. Stable Anion-Derived Solid Electrolyte Interphase in Lithium Metal Batteries. Angew.Chem. Int. Ed. 2021, 60, 22683–22687. 10.1002/anie.202107732. 34399018

[ref62] Jiang L. L.; Yan C.; Yao Y. X.; Cai W.; Huang J. Q.; Zhang Q. Inhibiting Solvent Co-Intercalation in a Graphite Anode by a Localized High-Concentration Electrolyte in Fast-Charging Batteries. Angew.Chem. Int. Ed. 2021, 60, 3402–3406. 10.1002/anie.202009738. 33107707

[ref63] Okoshi M.; Chou C. P.; Nakai H. Theoretical Analysis of Carrier Ion Diffusion in Superconcentrated Electrolyte Solutions for Sodium-Ion Batteries. J. Phys. Chem. B 2018, 122, 2600–2609. 10.1021/acs.jpcb.7b10589. 29433319

[ref64] Yu Z.; Balsara N. P.; Borodin O.; Gewirth A. A.; Hahn N. T.; Maginn E. J.; Persson K. A.; Srinivasan V.; Toney M. F.; Xu K.; Zavadil K. R.; Curtiss L. A.; Cheng L. Beyond Local Solvation Structure: Nanometric Aggregates in Battery Electrolytes and Their Effect on Electrolyte Properties. ACS Energy Lett. 2022, 7, 461–470. 10.1021/acsenergylett.1c02391.

