# Supplementary material for: Significance of Antisolvents on Solvation Structures Enhancing Interfacial Chemistry in Localized High-Concentration Electrolytes
Source: ACS Cent Sci. 2022 Aug 31;8(9):1290–8. doi: 10.1021/acscentsci.2c00791 (PMC9523775; doi:10.1021/acscentsci.2c00791)

oc-2022-00791k.R1

Name: Peer Review Information for "The significance of antisolvents on solvation structures enhancing interfacial chemistry in localized high-concentration electrolytes"

First Round of Reviewer Comments

Reviewer: 1

Comments to the Author

Recommendation: minor revision

Localized concentrated electrolytes show attractive compatibility with new electrode materials and new battery chemistries. However, the unclear understanding of the effects of antisolvent makes a rational design of this kind of electrolyte still a problem. This paper reports that the antisolvent in the localized concentrated electrolyte can decrease the binding energy between Li+□□□solvent and Li+□□□anion, which facilitates Li+ ion transport and brings changes to the electrochemical oxidation/reduction. These new understandings of localized concentrated electrolytes open a new way to optimize the solvation sheath to get robust SEI/CEI. It will attract wide attention for scientists in the field of batteries and applied chemistry. There are some suggestions and questions for the authors:

1. Please briefly summarized the understanding of the antisolvents' influence on the solvation structures, and related characterization methods from other work in the introduction and highlight the differences and significance of this work.

Other reports and methods are suggested but not limited as: J. Electrochem. Soc., 2021, DOI:10.1149/1945-7111/abd60e; Angewandte Chemie International Edition, 2022, DOI: 10.1002/anie.202205045; Angew. Chem. Int. Ed. 2021. DOI: 10.1002/anie.202101627; Angewandte Chemie International Edition, 2022.DOI:10.1002/anie.202207018; NMR, charge transfer resistance by EIS, etc.

2. In the conclusion part, is the binding energy between Li+---solvent or Li+---anion decreases with the addition of antisolvent? Please elaborate more. The desolvation energy is also mentioned, please elaborate more on its relationship with the binding energy.

3. The FTIR spectra without lithium salt are suggested to be provided to exclude the influence of the antisolvents (TFETFE and TTE) on DMC.

4. Please make careful modifications to English/wording. For example, on Page 3 Line 31 says "favor reducing …and the reduction stability of FSI- …", please make sure to use some more straight forwarding wording instead of "reducing the reduction".

5. Please add the concentration of TFETFE in Page 6, Line 22

Reviewer: 2

Comments to the Author

Localized concentrated electrolytes enable stable interphase layer for cathode/anode due to their unique solvation structure. Generally, the basic function of the anti-solvents in these electrolytes is generally thought to simply dilute the solvated clusters. This MS reported that antisolvent can also play roles to the solvation sheath through interactions with the solvated solvent molecules. This finding enriches the cognition on the localized concentrated electrolytes, delighting a rational strategy to tune the solvation structure to engineer the surface layer of electrode material. It will receive wide attention of researchers in the field of electrochemistry and solution chemistry. However, there still be some questions, as shown below. I recommend acception after minor revision.

1: FTIR (Figure 1 and Figure 2) is used to identify the interaction between DMC and antisolvents. As the change in the spectra may also be caused by the absorption of antisolvents, the FTIR spectra of antisolvents need to be provided to make solid conclusion.

2: Please check the MS carefully to avoid unnecessary errors. For example, in page 13, figure 4, “FSI” would be “FSI-”. In page 16, “favourable” would be “favorable”. “Figure 4. The population of Li+ solvation structures in HCE and LHCEs extract from MD simulation.” Where the “extract” should be past tense “extracted”.

3: The radial distribution function shows the distance between two atoms in a system. In Figure 3, one atom and a molecule are represented. Please double check the result and give clear illustration about it.

Author's Response to Peer Review Comments:

Dear Editor and reviewers

We are grateful to reviewers for reviewing our paper. We particularly appreciate reviewers for the positive comments and many detailed useful suggestions for revision. We have very carefully gone through all these technical points and addressed all of them in the revised version.

We addressed the reviewers' comments point by point below.

Sincerely yours

Xiangming He

Reviewer: 1

Recommendation: Publish in ACS Central Science after minor revisions noted.

Comments:
Recommendation: minor revision

Localized concentrated electrolytes show attractive compatibility with new electrode materials and new battery chemistries. However, the unclear understanding of the effects of antisolvent makes a rational design of this kind of electrolyte still a problem. This paper reports that the antisolvent in the localized concentrated electrolyte can decrease the binding energy between Li^+…solvent and Li^+…anion, which facilitates Li+ ion transport and brings changes to the electrochemical oxidation/reduction. These new understandings of localized concentrated electrolytes open a new way to optimize the solvation sheath to get robust SEI/CEI. It will attract wide attention for scientists in the field of batteries and applied chemistry. There are some suggestions and questions for the authors:

1. Please briefly summarized the understanding of the antisolvents' influence on the solvation structures, and related characterization methods from other work in the introduction and highlight the differences and significance of this work.
Other reports and methods are suggested but not limited as: J. Electrochem. Soc., 2021, DOI:10.1149/1945-7111/abd60e; Angewandte Chemie International Edition, 2022, DOI: 10.1002/anie.202205045; Angew. Chem. Int. Ed. 2021. DOI: 10.1002/anie.202101627; Angewandte Chemie International Edition, 2022.DOI:10.1002/anie.202207018; NMR, charge transfer resistance by EIS, etc.

Response: We thank the reviewer very much and these are important for us to understand the role of antisolvent. Now, they are incorporated into the revised manuscript in the introduction section as Ref. 30 -33 on page 4, line 22.
The revised discussion is as follows: "Recently, many works have demonstrated that antisolvents do not participate in the solvation sheath within Li^+ by MD simulations

and spectroscopy. Nevertheless, the influence of antisolvent on the solvation structures has been observed[30-33]. For instance, Li et al. have utilized the Raman spectra, nuclear magnetic resonance (NMR) spectra, and MD simulations to study the solvation structures of Na^{+}[32]. They find that the higher 1*H*,1*H*,5*H*-octafluoropentyl-1,1,2,2-tetrafluoroethyl ether (OTE) additions can enhance the coordination of FSI^{-} anions with Na^{+}, due to the dissolution of 1,2-dimethoxyethane (DME) in OTE. Huang et al. report that low dielectric environment afforded by antisolvent can enhance the interaction between anion and Li^{+} in DME-based LHCEs[30]. However, the mechanism of how an antisolvent acts on the solvation structure and changes the electrochemical reactivity of the solvation structure remains unclear and inadequate."

We also rewrite the important conclusions and simply make the significance of this work clearer in introduction section on page 5, line 9. As follows: "Specifically, antisolvents affect the solvation structure through intermolecular interactions with the solvent and consequently optimize the electrochemical stability of electrolyte components. The degree of influence depends on the type of antisolvent. In addition, antisolvents enable the Li^{+} desolvation process and promote the Li^{+} transport in LHCEs."

2. In the conclusion part, is the binding energy between Li+---solvent or Li+---anion decreases with the addition of antisolvent? Please elaborate more. The desolvation energy is also mentioned, please elaborate more on its relationship with the binding energy.

Response: We appreciate the reviewer's valuable suggestions. As pointed out by the reviewer, the binding energy between Li^{+}···solvent or Li^{+}···anion is decreased with the addition of antisolvent. We have added more discussions required on the binding energy between Li^{+}···solvent or Li^{+}···anion results in the revised manuscript (page 11, line 20). As follows: "The results show that the binding energy of the Li^{+}-DMC complex decreases from -0.998 eV to approximately -0.79 eV, while the binding energy of Li^{+}-FSI^{-} complex decreases from -1.965 to -1.16 eV with the addition of antisolvents. Noticeably, the binding energy of Li^{+}-FSI^{-} complex is decreased more than that of Li^{+}-DMC complex, indicating that antisolvents would prevent the association of FSI^{-} and Li^{+}."

In the conclusion part, we elaborate on the result of binding energy by using "The binding energies of the Li^{+}···DMC and Li^{+}···FSI^{-} interactions are decreased with the addition of antisolvent compared with the superconcentrated electrolytes, suggesting that the antisolvents could help to lower the desolvation energy and facilitate interface kinetics in the electrochemical reaction." (page 18, line 15) instead of "The reduced binding energies of the Li^{+}···DMC and Li^{+}···FSI^{-} interactions, when compared with the superconcentrated electrolytes, suggested that the antisolvents may help to lower the desolvation energy and facilitate interface kinetics in the electrochemical reaction.".

We have updated the relationship between desolvation energy and binding energy in the revised manuscript on page 12, line 3, just as follows:

"For desolvation energy, it is important for the desolvation process and the weaker ion desolvation energy is beneficial for ion diffusion between the electrolyte/electrode interface and improves the rate performance of batteries as described in Ref. 53-56. In addition, the positive correlation between desolvation energy and binding energy has been demonstrated by many works in Ref. 57-60."

3. The FTIR spectra without lithium salt are suggested to be provided to exclude the influence of the antisolvents (TFETFE and TTE) on DMC.

Response: This question is appreciated very much. Now, the FTIR spectra of antisolvents are provided in Figure 2a, and the related discussion can be found in the revised manuscript on page 10, line 7, as follows: "Moreover, the FTIR spectra of antisolvents are also provided in Figure 2a. It shows that the FTIR spectra of TFETFE and TTE almost no absorption at ten times magnification in the region around 1710 - 1800 cm^{-1}. These results indicate that the antisolvents do not interfere with the accuracy of the results."

4. Please make careful modifications to English/wording. For example, on Page 3 Line 31 says "favor reducing …and the reduction stability of FSI- …", please make sure to use some more straight forwarding wording instead of "reducing the reduction".

Response: We have carefully checked and modified the manuscript. The sentence mentioned by the reviewer is revised as follows (Page 2, Line 13):
"Furthermore, antisolvents also favor reducing the lowest unoccupied molecular orbital (LUMO) energy level of the solvated clusters and FSI^- anions show a decreased reduction stability."

5. Please add the concentration of TFETFE in Page 6, Line 22

Response: Thank you for underlining this deficiency. Now, the concentration of TFETFE is added on page 5, line 18 as "1,1,2,2-Tetrafluoroethyl-2,2,2-trifluoroethyl ether (TFETFE) (99.8%)"

Reviewer: 2

Recommendation: Publish in ACS Central Science after minor revisions noted.

Comments:
Localized concentrated electrolytes enable stable interphase layer for cathode/anode

due to their unique solvation structure. Generally, the basic function of the anti-solvents in these electrolytes is generally thought to simply dilute the solvated clusters. This MS reported that antisolvent can also play roles to the solvation sheath through interactions with the solvated solvent molecules. This finding enriches the cognition on the localized concentrated electrolytes, delighting a rational strategy to tune the solvation structure to engineer the surface layer of electrode material. It will receive wide attention of researchers in the field of electrochemistry and solution chemistry. However, there still be some questions, as shown below. I recommend acception after minor revision.

1: FTIR (Figure 1 and Figure 2) is used to identify the interaction between DMC and antisolvents. As the change in the spectra may also be caused by the absorption of antisolvents, the FTIR spectra of antisolvents need to be provided to make solid conclusion.

Response: This is an important question. Actually, reviewer 1 also made a similar comment: "*The FTIR spectra without lithium salt are suggested to be provided to exclude the influence of the antisolvents (TFETFE and TTE) on DMC.*". We thank both reviewers for pointing out the limitation of our results. The FTIR data of antisolvents and a discussion about FTIR have been provided in the revised manuscript.
on page 10, line 7, as follows: "Moreover, the FTIR spectra of antisolvents are also provided in Figure 2a. It shows that the FTIR spectra of TFETFE and TTE almost no absorption at ten times magnification in the region around 1710 - 1800 cm^{-1}. These results indicate that the antisolvents do not interfere with the accuracy of the results."

2: Please check the MS carefully to avoid unnecessary errors. For example, in page 13, figure 4, "FSI" would be "FSI^-". In page 16, "favourable" would be "favorable". "Figure 4. The population of Li^+ solvation structures in HCE and LHCEs extract from MD simulation." Where the "extract" should be past tense "extracted".

Response: We thank the reviewer very much. We checked the manuscript carefully and made corrections to errors mentioned by the reviewer on page15, Figure 4 and on page 18, line 5.
In addition, we have checked the whole manuscript carefully to avoid other incorrect expressions as well. We have fixed the wrong way of writing about "FSI^-" and "Li^+" in Figure 2d, Figure 5, Figure 6, Table 2, and on page 14.

3: The radial distribution function shows the distance between two atoms in a system. In Figure 3, one atom and a molecule are represented. Please double check the result and give clear illustration about it.

Response: This comment is appreciated very much. Here, FSI^- in Figure 3(b, d, f)

should be anion, and we have fixed the error in the revised manuscript on page 12, Figure 3. The modified diagram as follows:

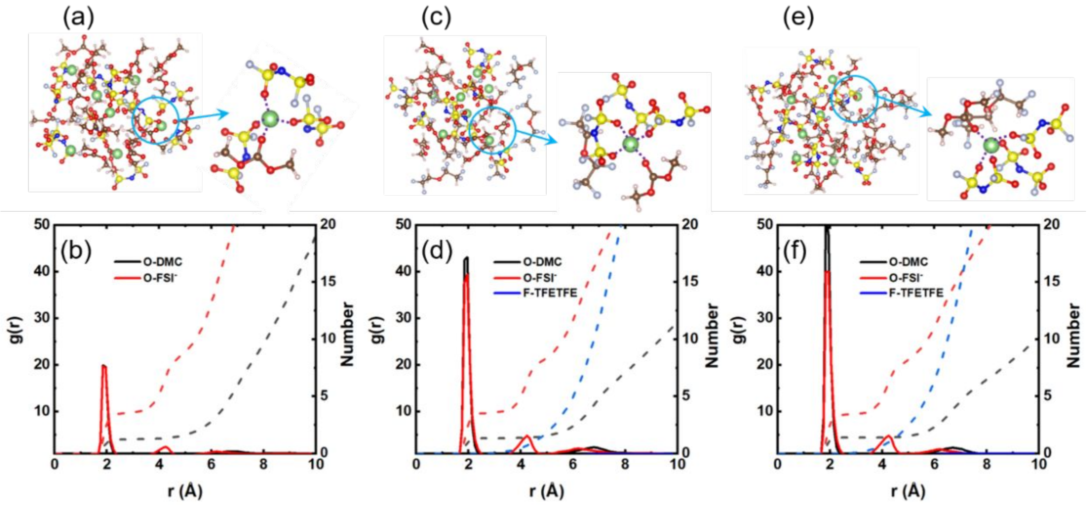

Supplement: Supplementary file 1 — oc2c00791_si_001.pdf [file oc2c00791_si_001.pdf]
